# Association of different biomarkers of renal function with D-dimer levels in patients with type 1 diabetes mellitus (renal biomarkers and D-dimer in diabetes)

**DOI:** 10.20945/2359-3997000000003

**Published:** 2018-01-01

**Authors:** Caroline Pereira Domingueti, Rodrigo Bastos Fóscolo, Luci Maria S Dusse, Janice Sepúlveda Reis, Maria das Graças Carvalho, Karina Braga Gomes, Ana Paula Fernandes

**Affiliations:** 1 Universidade Federal de São João del-Rei Universidade Federal de São João del-Rei Campus Centro Oeste Dona Lindu Divinópolis MG Brasil Campus Centro Oeste Dona Lindu, Universidade Federal de São João del-Rei (UFSJ), Divinópolis, MG, Brasil; 2 Universidade Federal de Minas Gerais Universidade Federal de Minas Gerais Faculdade de Medicina Departamento de Clínica Médica Belo Horizonte MG Brasil Departamento de Clínica Médica, Faculdade de Medicina, Universidade Federal de Minas Gerais (UFMG), Belo Horizonte, MG, Brasil; 3 Universidade Federal de Minas Gerais Universidade Federal de Minas Gerais Faculdade de Farmácia Departamento de Análises Clínicas e Toxicológicas Belo Horizonte MG Brasil Departamento de Análises Clínicas e Toxicológicas, Faculdade de Farmácia, Universidade Federal de Minas Gerais (UFMG), Belo Horizonte, MG, Brasil; 4 Instituto de Ensino e Pesquisa da Santa Casa de Belo Horizonte Departamento de Endocrinologia e Metabologia Belo Horizonte MG Brasil Departamento de Endocrinologia e Metabologia, Instituto de Ensino e Pesquisa da Santa Casa de Belo Horizonte, Belo Horizonte, MG, Brasil

**Keywords:** D-Dimer, type 1 diabetes mellitus, cystatin C, creatinine, albuminuria

## Abstract

**Objective:**

This study aimed to evaluate the association between different renal biomarkers with D-Dimer levels in diabetes mellitus (DM1) patients group classified as: low D-Dimer levels (< 318 ng/mL), which included first and second D-Dimer tertiles, and high D-Dimer levels (≥ 318 ng/mL), which included third D-Dimer tertile.

**Materials and methods:**

D-Dimer and cystatin C were measured by ELISA. Creatinine and urea were determined by enzymatic method. Estimated glomerular filtration rate (eGFR) was calculated using CKD-EPI equation. Albuminuria was assessed by immunoturbidimetry. Presence of renal disease was evaluated using each renal biomarker: creatinine, urea, cystatin C, eGFR and albuminuria. Bivariate logistic regression analysis was performed to assess which renal biomarkers are associated with high D-Dimer levels and odds ratio was calculated. After, multivariate logistic regression analysis was performed to assess which renal biomarkers are associated with high D-Dimer levels (after adjusting for sex and age) and odds ratio was calculated.

**Results:**

Cystatin C presented a better association [OR of 9.8 (3.8–25.5)] with high D-Dimer levels than albuminuria, creatinine, eGFR and urea [OR of 5.3 (2.2–12.9), 8.4 (2.5–25.4), 9.1 (2.6–31.4) and 3.5 (1.4–8.4), respectively] after adjusting for sex and age. All biomarkers showed a good association with D-Dimer levels, and consequently, with hypercoagulability status, and cystatin C showed the best association among them.

**Conclusion:**

Therefore, cystatin C might be useful to detect patients with incipient diabetic kidney disease that present an increased risk of cardiovascular disease, contributing to an early adoption of reno and cardioprotective therapies.

## INTRODUCTION

Diabetic kidney disease is defined as a progressive increase in urinary albumin excretion (UAE), leading to glomerular filtration declining and, eventually, renal failure (1). It is the most important cause of end-stage renal disease, is an independent risk factor for cardiovascular disease and is responsible for increased mortality. It is estimated that nearly 30% of patients with diabetes develop renal disease (2,3).

Several biomarkers can be used to evaluate renal function in patients with diabetes, such as creatinine, urea, glomerular filtration rate (GFR), UAE and cystatin C. Creatinine is derived from metabolism of creatine and phosphocreatine of muscle cells, while urea is the major nitrogenous metabolite derived from the degradation of proteins (4,5). However, various factors can influence their levels aside from renal disease; therefore, the estimative of GFR is more often used in clinical practice (4,5). UAE is an important biomarker of renal injury, which is used for the diagnosis and prognosis of diabetic kidney disease, since it enables early detection of renal parenchyma injury (4,6). Cystatin C is a low molecular weight protein synthesized by all nucleated cells, whose function is to regulate cysteine proteases. It has been shown to be very promising in detecting early stages of renal disease in diabetic patients (6,7).

D-Dimer is a specific degradation product of crosslinked fibrin clots. It is a classic hypercoagulability biomarker, useful in the diagnosis of thromboembolic events (8). There is an association between D-Dimer levels with the development of atherothrombosis and cardiovascular complications in patients with diabetes, indicating that D-Dimer can be useful in evaluating the risk of cardiovascular disease in these patients (8-10). D-Dimer levels also increase with the progression of renal disease in patients with diabetes, indicating that hypercoagulability could be a link between diabetic kidney disease and the increased risk of cardiovascular outcomes (11-14).

A biomarker that is capable of detecting early stages of renal function decline and, therefore, could be simultaneously associated with a higher hypercoagulability status could be of great value. This is because it could be useful for detecting patients with incipient diabetic kidney disease that present an increased risk of cardiovascular disease, contributing to an early adoption of reno and cardioprotective therapies and, consequently, to a reduction of mortality.

Therefore, this study aimed to evaluate the association between D-Dimer levels and different biomarkers to assess the relationship between hypercoagulability and renal disease in patients with type 1 diabetes mellitus (DM1).

## MATERIALS AND METHODS

The study was performed in accordance with the 2000 Declaration of Helsinki. It was approved by the Research Ethics Committee of *Universidade Federal de Minas Gerais* (CAAE – 0392.0.203.000-11), and informed consent was obtained from all participants.

Clinical records of 240 consecutive DM1 patients being assisted at Endocrinology Ambulatories of the Hospital das Clínicas and Santa Casa de Misericórdia of Belo Horizonte, Brazil, from November 2011 to September 2012, were analyzed. After the application of exclusion criteria, 125 patients with clinical and laboratorial diagnosis of DM1 (15), 18 to 60 years of age, were selected for this study. Criteria of exclusion consisted of hepatic disease, alcoholism, coagulation or hemostatic abnormalities, malignant diseases, acute infectious, history of kidney transplantation, pregnancy and hemodialysis. Data regarding age, sex, weight, height, time of diagnosis of DM1, use of antihypertensive, statin and acetylsalicylic acid were obtained from medical records.

Serum creatinine and urea were determined using an enzymatic method, serum albumin was assessed using a colorimetric method and HbA1c was determined through an immunoturbidimetric method, using dry chemistry technology. Cystatin C and D-Dimer were measured by ELISA. UAE was determined in urine samples collected after at least 4 hours of urinary retention, and urinary albumin was normalized by urinary creatinine. Urinary albumin was evaluated using an immunoturbidimetric method and urinary creatinine was assessed using an enzymatic method, using dry chemistry technology. UAE ≥ 30 mg/g of creatinine was confirmed in two out of three occasions during a period between three and six months, and the median was calculated (6). The estimated glomerular filtration rate (eGFR) was calculated using the CKDEPI equation (16).

Statistical analysis was performed using SPSS software v. 20.0. Patients were divided into tertiles based on D-Dimer levels and were classified into two groups: low D-Dimer levels (< 318 ng/mL), which included first and second D-Dimer tertiles, and high D-Dimer levels (≥ 318 ng/mL), which included third D-Dimer tertile (17). The Shapiro-Wilk test was used to test the normality of the variables. Data normally distributed were expressed as mean ± SD and were compared using ANOVA and a t-est. Data not normally distributed were expressed as median (percentiles 25%-75%) and were compared using the Kruskal-Wallis H test and Mann-Whitney U test. Categorical variables were expressed as frequencies and compared using a chi-square test (χ^2^). The presence of renal disease was evaluated using each renal biomarker; these were dichotomized using cutoff of ≥ 1.3 mg/dL, ≥ 40 mg/dL, ≥ 0.92 mg/L and ≥ 30 mg/g, for creatinine, urea, cystatin C and UAE, respectively (18-20). Bivariate logistic regression analysis was performed to assess which dichotomized renal biomarkers are associated with high D-Dimer levels and an odds ratio was calculated. Multivariate logistic regression analysis was performed to assess which dichotomized renal biomarkers are associated with high D-Dimer tertiles after adjusting for sex and age, and an odds ratio was calculated. The correlation between non-categorized renal biomarkers and D-Dimer levels were evaluated using the Spearman Correlation. Differences were considered significant when p ≤ 0.05.

## RESULTS

The characteristics and clinical variables of the DM1 patients included in this cross-sectional study are presented in Table 1.

**Table 1:** Characteristics of patients with diabetes classified according to D-Dimer levels

	Low D-Dimer Group	High D-Dimer Group	p
Number of individuals (n)	82	43	
Age (years)	32 (24 – 37)	35 (30 – 45)*	0.003
Sex/male (n, %)	37 (45.1)	8 (18.6)*	0.003
BMI (kg/m^2^)	24 ± 3	23 ± 3	NS
Time of diagnosis (years)	18 ± 8	20 ± 6	NS
Use of antihypertensive (n, %)	44 (53.7)	36 (83.7)*	0.001
Use of statin (n, %)	22 (26.8)	18 (41.9)	NS
Use of AAS (n, %)	10 (12.2)	11 (25.6)	NS
HbA1c (%)	8.5 (7.5 – 9.8)	8.4 (7.6 – 8.4)	NS
Creatinine (mg/dL)	0.81 (0.66 – 0.92)	1.02 (0.71 – 1.45)*	0.001
eGFR (mL/min/1.73 m^2^)	112 (91 – 123)	76 (43 – 104)*	< 0.001
Urea (mg/dL)	31 ± 7	42 ± 17*	< 0.001
Albumin (g/dL)	4.1 ± 0.4	3.8 ± 0.4*	0.006
Cystatin C (mg/L)	0.74 (0.64 – 0.85)	1.11 (0.86 – 1.97)*	< 0.001
UAE (mg/g of creatinine)	8 (4 – 18)	44 (6 – 157)*	0.004
D-Dimer (ng/mL)	191 (134 – 233)	484 (381 – 639)*	< 0.001

Normally-distributed data were expressed as mean ± SD and compared by ANOVA and T test. Not normally distributed data were expressed as median (percentiles 25% – 75%) and compared by the Kruskal-Wallis H test and Mann-Whitney U test, followed by Bonferroni correction. Categorical variables were expressed as frequencies n (%) and compared using the chi-square test (χ^2^).

* p < 0.05 for high D-Dimer group compared to low D-Dimer group.

NS: not significant. BMI: body mass index. UAE: urinary albumin excretion. AAS: acetylsalicylic acid.

Patients with high D-Dimer levels were older (p = 0.003) and presented an increased frequency of antihypertensive use than those with low D-Dimer levels (p = 0.001). The frequency of males was decreased in the high D-Dimer group compared to the low D-Dimer group (p = 0.003). There were no significant differences among groups regarding BMI, time of diagnosis, HbA1c levels, use of statin and use of AAS. Reduced serum albumin was observed in patients of the high D-Dimer group when compared to the low D-Dimer group (p = 0.006). Patients with high D-Dimer levels presented more increased levels of creatinine, eGFR, urea, cystatin C and UAE than patients with low D-Dimer levels (p = 0.001, p < 0.001, p < 0.001, p < 0.001 and p = 0.004, respectively).

Bivariate logistic regression analysis has demonstrated that patients with cystatin C ≥ 0.92 mg/L showed a better association with high D-Dimer levels [OR of 9.0 (3.8–21.1)], than patients with UAE ≥ 30 mg/g, creatinine ≥ 1.3 mg/dL, eGFR < 60 mL/min/1.73 m^2^ and urea ≥ 40 mg/dL [OR of 5.0 (2.2–11.4), 5.3 (2.1–13.3), 6.0 (2.3–15.7) and 3.3 (1.5–7.3), respectively] (Table 2). A multivariate logistic regression analysis has shown that the association between all renal biomarkers and high D-Dimer levels remained, even after adjusting for sex and age (Table 2). After the adjustment for sex and age, patients with cystatin C ≥ 0.92 mg/L remained, presenting a better association with high D-Dimer levels [OR of 9.8 (3.8–25.5)] than patients with UAE ≥ 30 mg/g, creatinine ≥ 1.3 mg/dL, eGFR < 60 mL/min/1.73 m^2^ and urea ≥ 40 mg/dL [OR of 5.3 (2.2–12.9), 8.4 (2.5–25.4), 9.1 (2.6–31.4) and 3.5 (1.4–8.4), respectively].

**Table 2:** Association between renal biomarkers and high D-Dimer levels

Variable	Odds ratio (95% confidence interval) unadjusted	p*	Odds ratio (95% confidence interval) adjusted for sex and age	p*
Creatinine ≥ 1.3 mg/dL	5.303 (2.106 – 13.357)	< 0.001	8.374 (2.464 – 28.459)	< 0.001
eGFR < 60 mL/min/1.73 m^2^	6.048 (2.335 – 15.666)	< 0.001	9.110 (2.643 – 31.408)	< 0.001
Urea ≥ 40 mg/dL	3.340 (1.535 – 7.271)	< 0.001	3.480 (1.433 – 8.453)	0.008
Cystatin C ≥ 0.92 mg/L	9.018 (3.853 – 21.109)	< 0.001	9.844 (3.796 – 25.527)	< 0.001
UAE ≥ 30 mg/g	5.042 (2.222 – 11.440)	< 0.001	5.285 (2.160 – 12.926)	< 0.001

Data was evaluated by bivariate and multivariate logistic regression analysis and are presented as odds ratio (95% confidence interval). NS = not significant.

* p < 0.05 for high D-Dimer group compared to low D-Dimer group.

**Figure 1:**
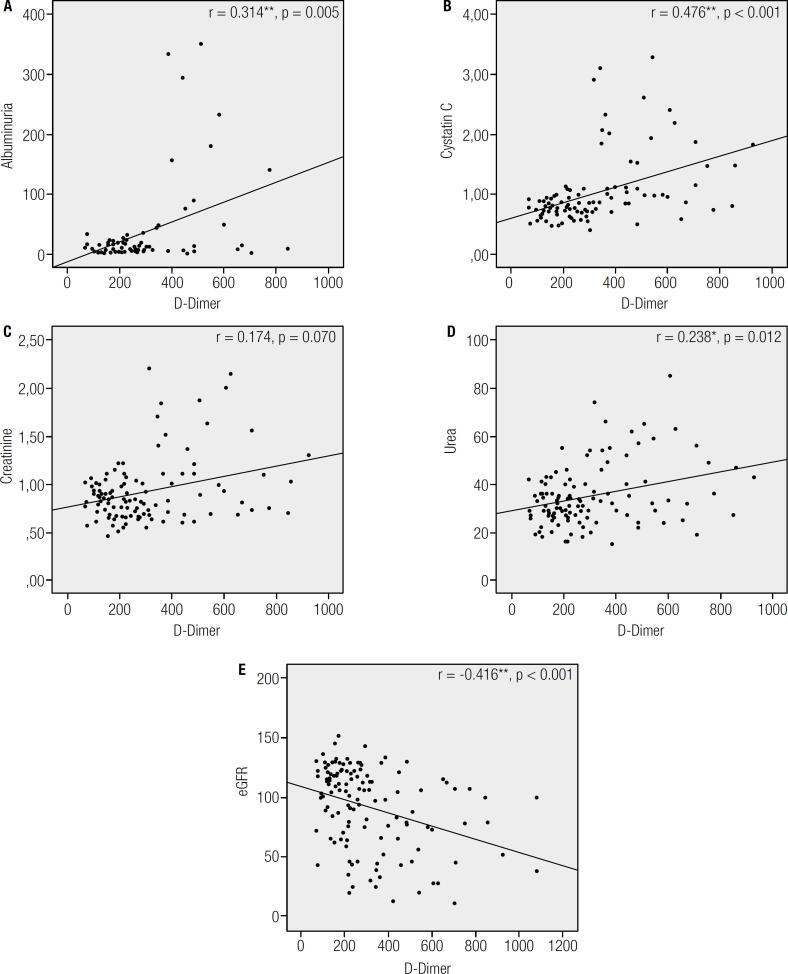
Spearman correlation of albuminuria (**A**), cystatin C (**B**), creatinine (**C**), urea (**D**) and (**E**) eGFR with D-Dimer levels. * Correlation is significant at the 0.05 level. ** Correlation is significant at the 0.01 level.

Cystatin C levels correlated better with D-Dimer levels (r = 0.476, p < 0.001) than other renal biomarkers (r = 0.174, p = 0.070 for creatinine; r = 0.238, p = 0.012 for urea; r = -0416, p < 0.001 for eGFR; r = 0.314, p = 0.005 for albuminuria) (Figure 1).

## DISCUSSION

Diabetic kidney disease is associated with an increased mortality, mainly due to cardiovascular outcomes (6). It has been demonstrated that the risk of cardiovascular death gradually increases with progressing stages of kidney disease (21,22). Therefore, simultaneous evaluation of early stages of renal function and hypercoagulability status using a unique biomarker would be of great value. Here, we have evaluated the association between different biomarkers of renal function with hypercoagulability status as assessed by D-Dimer levels in DM1 patients.

Some studies have found an association between increased D-Dimer levels and the presence of increased UAE levels and reduced eGFR in patients with diabetes (12-14,23). In this study, increased levels of different renal biomarkers, such as creatinine, urea, cystatin C, eGFR and UAE, were observed in patients with high D-Dimer levels. The association between renal dysfunction and increased levels of D-Dimer in DM1 patients may be explained by the increased synthesis of D-Dimer, but not by the reduced loss of D-Dimer in urine, since it has been shown that patients with diabetic kidney disease show higher urinary levels of D-Dimer than healthy individuals due to proteinuria (24). Proteinuria should also be responsible for the loss of important natural anticoagulant proteins, such as antithrombin, protein C and protein S, intensifying the hypercoagulability status and the production of D-Dimer (25).

It was verified that the decline of GFR also results in endothelial dysfunction and the release of the von Willebrand factor, which promotes platelet adhesion and aggregation and, consequently, microthrombi formation and increased D-Dimer levels (26). Endothelial dysfunction also impairs the activation of protein C, which depends on the endothelial protein C receptor and thrombomodulin, whose expression is reduced in damaged microvasculature, enhancing hypercoagulability status (27).

Haase and cols. (28) has demonstrated that D-Dimer plasma levels are higher in older adults and in females, which was also found in this study. These findings could be explained by the development of age-related changes in microcirculation and blood coagulation, which contribute to generate a hypercoagulability status and a gradual increase of D-Dimer levels with aging, as well as by the use of hormonal contraceptives in most of women. Such contraceptives can promote clotting mechanisms and increase D-Dimer levels (29,30). Increased frequency of antihypertensive use was observed in patients with high D-Dimer levels, which was expected, since these patients also showed an increased frequency of renal disease. Antihypertensive is commonly prescribed to patients with diabetes who have kidney disease to protect renal function (31). These patients also showed reduced serum albumin, which is in accordance to the increased UAE.

The relationship between increased UAE levels with a higher risk of cardiovascular disease in DM1 patients has been demonstrated by several authors (21,32,33). After the onset of proteinuria, median survival is only about seven years, and this increased mortality is mainly due to cardiovascular death rather than renal failure (34). High levels of serum creatinine and reduced eGFR have also been demonstrated to be indicative of progressive cardiovascular disease among diabetic patients (35,36), and increased levels of cystatin C have been associated with the development of cardiovascular events (37-40). Some authors have even shown that cystatin C is a stronger predictor of cardiovascular outcomes in patients with diabetes and elderly adults than creatinine and eGFR (39,40).

In agreement in this study, cystatin C presented a better association – when assessed by odds ratio analysis – with higher D-Dimer levels than urea, creatinine, eGFR and UAE, after adjusting for sex and age, which are variables that can influence D-Dimer levels (28). Cystatin C levels also presented a better correlation with D-Dimer levels than other renal biomarkers. These results suggest that cystatin C presents a better association with hypercoagulability status than other renal biomarkers and that it might be able to detect hemostatic changes that are not completely captured by measurements of urea, creatinine, eGFR and UAE. Cystatin C has been demonstrated to be a better biomarker to detect early stages of chronic kidney disease than creatinine and eGFR (41,42). This could partially explain the better association of this renal biomarker with higher D-Dimer levels, since cystatin C could detect a decline of renal function, and consequently a hypercoagulability state, that is not detected by other biomarkers. However, further longitudinal studies that directly assess the development of cardiovascular disease are still necessary to confirm the superiority of cystatin C to predict this risk in comparison to other renal biomarkers.

Some studies have reported that arteries with atherosclerosis contain more cysteine proteases than normal arteries, which may contribute to the degradation of atherosclerotic plaque (43,44). Cystatin C is a protein responsible for inhibit cysteine proteases and is a biomarker able to detect early stages of chronic kidney disease (41,42). Therefore, patients with renal disease present high levels of cystatin C, which may inhibit proteases that promote the degradation of atherosclerotic plaque. This contributes to the development of atherosclerosis and cardiovascular disease and explains why cystatin C is the renal biomarker that presents a better association with hypercoagulability status. However, this hypothesis should be further evaluated.

In conclusion, all renal biomarkers showed a good association with D-Dimer levels and, consequently, with hypercoagulability status. However, cystatin C showed the best association among them. These findings suggest that cystatin C might present an important utility to simultaneously evaluate renal function decline and the hypercoagulability status in DM1 patients.
